# Imaging for Small UAV-Borne FMCW SAR

**DOI:** 10.3390/s19010087

**Published:** 2018-12-27

**Authors:** Xianyang Hu, Changzheng Ma, Ruizhi Hu, Tat Soon Yeo

**Affiliations:** 1 Department of Electrical and Computer Engineering, National University of Singapore, 117583 Singapore, Singapore; elehuru@nus.edu.sg (R.H.); eleyeots@nus.edu.sg (T.S.Y.); 2 MooVita Pte Ltd., 369977 Singapore, Singapore; eeczma@gmail.com

**Keywords:** UAV SAR, FMCW, intra-pulse motion, Squinted Azimuth-dependent PGA

## Abstract

Unmanned aerial vehicle borne frequency modulated continuous wave synthetic aperture radars are attracting more and more attention due to their low cost and flexible operation capacity, including the ability to capture images at different elevation angles for precise target identification. However, small unmanned aerial vehicles suffer from large trajectory deviation and severe range-azimuth coupling due to their simple navigational control and susceptibility to air turbulence. In this paper, we utilize the squint minimization technique to reduce this coupling while simultaneously eliminating intra-pulse motion-induced effects with an additional spectrum scaling. After which, the modified range doppler algorithm is derived for second order range compression and block-wise range cell migration correction. Raw data-based motion compensation is carried out with a doppler tracker. Squinted azimuth dependent phase gradient algorithm is employed to deal with azimuth dependent parameters and inexact deramping, with minimum entropy-based autofocusing algorithms. Finally, azimuth nonlinear chirp scaling is used for azimuth compression. Simulation and real data experiment results presented verify the effectiveness of the above signal processing approach.

## 1. Introduction

Different from optical sensors, synthetic aperture radar (SAR) is able to obtain images of different characteristics, and work at night and in all weather conditions. However, traditional satellite-bore and air-bore SAR systems are either inflexible for time-critical observing missions or operationally uneconomic. Unmanned aerial vehicle (UAV) borne SAR, being small-sized, easy to launch and remotely controllable, is thus a good choice for many applications, such as fire impact assessment, search and rescue operation, and civil infrastructure inspection missions.

It has long been a hot research topic for SAR system design to meet the size, weight and power requirements of small-sized platforms. To minimize hardware requirements, Frequency Modulated Continuous Wave (FMCW) waveform is usually used. Unfortunately FMCW operation violates the stop-and-go assumption commonly used for pulsed radar systems. It also introduces additional Doppler shifts to the spectrum and range profiles as well as range defocusing and high order phase errors. Similar to Range Cell Migration (RCM) and second order range defocusing problems, these effects tend to be more severe as squint angle increases. Most of the papers correct range profile shift only, leaving range defocusing and high order phase errors uncompensated [1]. Others take all of them into consideration by exploiting analytical two-dimensional spectrum, but resulting in much too complicated signal formulae, making them not straight-forward for subsequent analysis [2].

As is well known, steering radar beam from zero Doppler direction can provide squint angle- dependent scattering information, and also help to fill up shadow areas. It also offers flexibility for tactical purposes. On the other hand, it imposes requirements for higher radar pulse repetition frequency (PRF) and more efforts in signal processing. To deal with the intensified range-azimuth coupling in squint cases, and avoid computational efficiency degradation most time domain algorithms have experienced [3,4,5,6,7,8], several frequency domain algorithms have been devised and modified, such as ω−k [9], RD with Second-order Range Compression (SRC) [10], Non-linear Chirp Scaling with range dependent SRC taken into consideration [11], just to name a few. However, these algorithms all work with a skewed spectrum, which can easily lead to spectrum aliasing in high squint angle or wide beam cases. Another strategy is to exploit the squint minimization technique [12], which deforms the skewed spectrum into a regular one and handles the side effects it brings about in later processing, so that PRF can be largely reduced and system complexity can be controlled. The side effects consist mainly of azimuth dependent RCM and Doppler rate, and scatterers of different slant ranges residing in the same range gate, thus requiring further correction processing.

Due to its light weight and aerodynamic mechanisms, rotor-UAV is very susceptible to atmosphere influences and platform disturbances, making Motion Compensation (MoCo) indispensable. Furthermore, small UAVs cannot carry high accuracy Inertial Navigation System (INS) due to their limited load capacity. Thus it is imperative to extract motion information from the raw data in order to obtain highly focused UAV SAR image. However, with the motion errors further complicated by the previously mentioned side effects, even the state-of-art phase gradient algorithm (PGA) [13,14,15,16] may fail to extract the motion information accurately if directly applied. What’s more, as SAR operating frequency gets higher and higher, it will be harder and harder for motion compensation to be done, since even moderate deviations will lead to phase error of over several radians.

In this paper, we propose to deal with range-azimuth coupling using the squint minimization technique, which provides a more interpretable and near full solution to the intra-pulse motion issue. The azimuth dependent RCM is then rectified with a two-stage block-wise scheme, and finally, azimuth compression is carried out via Azimuth Nonlinear Chirp Scaling (ANCS) [12,17,18]. A Squinted Azimuth-dependent PGA (SAPGA) is being used to deal with the azimuth dependent Doppler parameter and inexact deramping problems in motion extraction. Autofocus is conducted with the proposed SAPGA and Minimum Entropy Algorithm (MEA) [19,20] based algorithms, after the severe platform instability had been partially stabilized with a Doppler tracker. The rest of the paper is organized as follows: squinted UAV-borne SAR geometry is described in Section 2, followed by imaging algorithm and motion compensation method in Section 3 and Section 4 respectively. Simulation and real data experiment results are given in Section 5, and finally conclusions are drawn in Section 6.

## 2. UAV-Borne SAR Geometry

**Figure 1:**
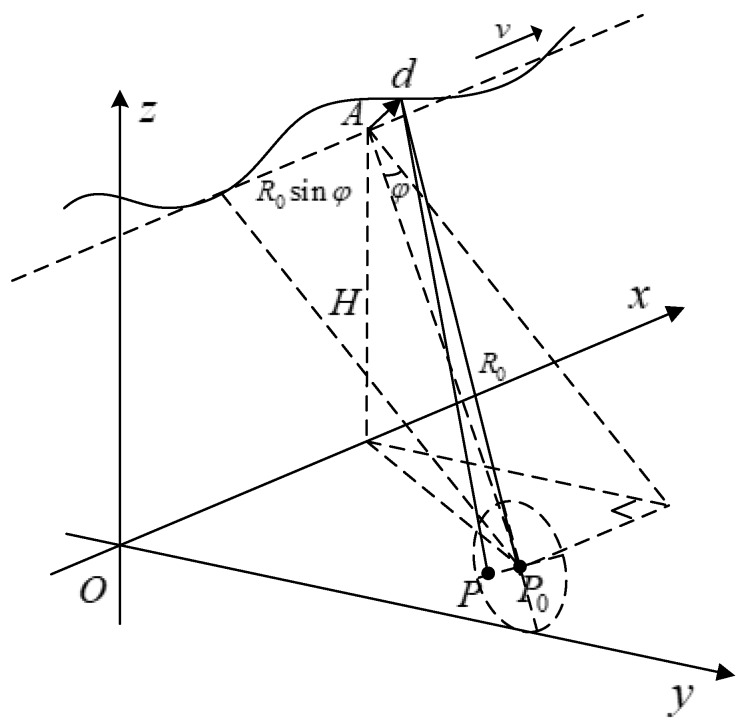
Radar platform motion geometry diagram.

In stripmap SAR imaging, the radar platform is assumed to be travelling in a uniform straight line, but due to platform perturbations and atmosphere turbulences, the platform may deviate from the ideal trajectory by a significant amount. A typical geometric diagram of the SAR system is depicted in Figure 1, where the dotted line is the ideal trajectory, and the solid sinusoid is the actual trajectory. Mathematically, for any scatterer $P\left(x_{n},\;y_{n},\;z_{n}\right)$ in the imaging scene, instantaneous slant range to the radar platform in its ideal trajectory $A\left(v_{x}t_{m},\;0,\;H\right)$ can be expressed as [9]:

(1) $$\begin{array}{c} \begin{array}{c} R\left(t_{m}\right)=∥P-A∥\\ =\sqrt{{\left(v_{x}t_{m}-x_{n}\right)}^{2}+{y_{n}}^{2}+{\left(H-z_{n}\right)}^{2}}\\ \approx \sqrt{{\left(R_{0}cos\varphi \right)}^{2}+v_{x}^{2}{\left(t_{m}+\frac{R_{0}sin\varphi -x_{n}}{v_{x}}\right)}^{2}} \end{array} \end{array}$$

where φ is the squint angle, and $R_{0}$ is the slant range at the aperture center.

The above radar distance model is derived under stop-and-go assumption valid for conventional pulsed radar, which assumes that the radar platform is stationary during the time it transmits a pulse and the time it receives its echo. For FMCW radar, effects of intra-pulse motion will be significant and affect image focusing, causing additional range shift, defocusing and phase artifacts. Thus the distance model has to be reconsidered [2]. Let FMCW signal be transmitted at instance $t_{m}$ and received at $t_{m}+t_{d}$, then $t_{d}$ satisfies:

(2) $$\begin{array}{cc} t_{d} & =\frac{\sqrt{{\left(R_{0}cos\varphi \right)}^{2}+v_{x}^{2}{\left(t_{m}+\frac{R_{0}sin\varphi -x_{n}}{v_{x}}\right)}^{2}}}{c}+\frac{\sqrt{{\left(R_{0}cos\varphi \right)}^{2}+v_{x}^{2}{\left(t_{m}+t_{d}+\frac{R_{0}sin\varphi -x_{n}}{v_{x}}\right)}^{2}}}{c}\\ & =\frac{1}{1-\frac{v_{x}^{2}}{c^{2}}}\left(\frac{2R\left(t_{m}\right)}{c}+\frac{2v_{x}^{2}}{c^{2}}\left(t_{m}+\frac{R_{0}sin\varphi -x_{n}}{v_{x}}\right)\right) \end{array}$$

further accounting for trajectory deviations with an initial displacement $d={\left(\Delta_{x},\Delta_{y},\Delta_{z}\right)}^{T}$, velocity error $v_{\Delta }={\left({v_{\Delta }}_{x},{v_{\Delta }}_{y},{v_{\Delta }}_{z}\right)}^{T}$, and acceleration error $\;a_{\Delta }={\left({a_{\Delta }}_{x},{a_{\Delta }}_{y},{a_{\Delta }}_{z}\right)}^{T}$, then:

(3) $$\begin{array}{c} \begin{array}{cc} R\left(t_{m}\right) & =∥P-\left(A+d+v_{\Delta }t_{m}+\frac{1}{2}a_{\Delta }{t_{m}}^{2}\right)∥\\ & \approx ∥P_{0}-A-\frac{\left(P_{0}-A\right)}{∥P_{0}-A∥}\cdot \left(d+v_{\Delta }t_{m}+\frac{1}{2}a_{\Delta }{t_{m}}^{2}\right)∥ \end{array} \end{array}$$

where $P_{0}=\left(0,0,0\right)$ is the scene center. The above approximation is made under far field and center beam approximations [21].

## 3. Imaging Algorithm

Suppose linear frequency modulated signal of chirp rate α is transmitted, then received signal after dechirp-on-receive processing with 0 derchirp delay can be written as:

(4) $$s\left(t,t_{m}\right)=rect\left(t-t_{d}\right)exp\left(-j2\pi \left(\left(f_{c}+\alpha t\right)t_{d}-\frac{\alpha }{2}{t_{d}}^{2}\right)\right)$$

where the second order term of $t_{d}$ is the Residual Video Phase (RVP) term, which can be easily removed [22].

Multiply with the squint minimization term $\;exp\left(jK_{r}v_{x}sin\varphi t_{m}\;\right)$, where $K_{r}=K_{c}+\Delta K=\frac{4\pi f_{c}}{c}+\frac{4\pi \alpha t}{c}$, $\beta =\frac{1}{1-\frac{v_{x}^{2}}{c^{2}}}$, replace $t_{m}$ with $t_{m}+t$, and take Fourier transform with respect to $t_{m}$ using principle of stationary phase, we have:

(5) $$\begin{array}{c} s\left(K_{r},K_{a}\right)=rect\left(\frac{K_{r}-K_{rc}}{4\pi \frac{\alpha }{\beta }}\right)rect\left(\frac{K_{a}-\beta K_{r}sin\varphi_{e}}{B_{a}}\right)\cdot \\ exp\left(\begin{array}{c} -jR_{0}cos\varphi \sqrt{{\left(\beta K_{r}\right)}^{2}-{\left(K_{a}-\beta K_{r}sin\varphi_{e}\right)}^{2}}\\ -j\left(K_{a}-\beta K_{r}sin\varphi_{e}\right)\left(\begin{array}{c} -v_{x}\frac{\Delta K\cdot c}{4\pi \alpha }\\ -sin\varphi R_{0}\\ +x_{n} \end{array}\right) \end{array}\right)exp\left(-j\phi \left(X^{s}\right)\;\right) \end{array}$$

in which $sin\varphi_{e}=\frac{sin\varphi }{\beta }-\frac{v_{x}}{c}$. For symbol simplification, $B_{a}=\beta K_{r}\left(\frac{sin\varphi_{max}}{\beta }-\frac{v_{x}}{c}\right)-\beta K_{r}\left(\frac{sin\varphi_{min}}{\beta }-\frac{v_{x}}{c}\right)$ is the azimuth spatial bandwidth, $\phi \left(X^{s}\right)=K_{r}\Delta R\left(X^{s}\right)$ accounts for trajectory errors, and

(6) $$X^{s}=-v_{x}\frac{\Delta K\cdot c}{4\pi \alpha }-R_{0}sin\varphi +x_{n}+\frac{\left(K_{a}-\beta K_{r}\left(\frac{sin\varphi }{\beta }-\frac{v_{x}}{c}\right)\right)R_{0}cos\varphi }{\sqrt{{\left(\beta K_{r}\right)}^{2}-{\left(K_{a}-\beta K_{r}\left(\frac{sin\varphi }{\beta }-\frac{v_{x}}{c}\right)\right)}^{2}}}$$

is the the point of stationary phase.

We note that, while $K_{r}$ is associated with a factor β in (5), (5) can be handled by replacing the squint minimizaiton term $\;exp\left(jK_{r}v_{x}sin\varphi_{e}t_{m}\;\right)$ with $\;exp\left(jK_{r}v_{x}sin\varphi_{\mathrm{adjust}}t_{m}\;\right)$, where $sin\varphi_{\mathrm{adjust}}=\beta sin\varphi +\beta \frac{v_{x}}{c}$. When followed by a slant range spectrum scaling, the square-root term is then identical to that of a pulsed SAR system. The impact on range resolution after slant range spectrum scaling shall be negligible since β is usually small, especially in UAV SAR.

Then $s\left(K_{r},K_{a}\right)$ can be rewritten as:

(7) $$\begin{array}{c} s\left(K_{r},K_{a}\right)=rect\left(\frac{K_{r}-K_{rc}}{4\pi \alpha }\right)rect\left(\frac{K_{a}-K_{r}sin\varphi }{B_{a}}\right)\cdot \\ exp\left(\begin{array}{c} -jR_{0}cos\varphi \sqrt{{K_{r}}^{2}-{\left(K_{a}-K_{r}sin\varphi \right)}^{2}}\\ -j\left(K_{a}-K_{r}sin\varphi \right)\left(-\Delta K\frac{v_{c}c}{4\pi \alpha \beta }-sin\varphi R_{0}+x_{n}\right) \end{array}\right)exp\left(-j\phi \left(X^{s}\right)\;\right) \end{array}$$

and

(8) $$\begin{array}{cc} X^{s}= & -v_{x}\frac{\Delta K\cdot c}{4\pi \alpha \beta }-R_{0}sin\varphi +x_{n}+\frac{\left(K_{a}-K_{r}sin\varphi \right)R_{0}cos\varphi }{\sqrt{{K_{r}}^{2}-{\left(K_{a}-K_{r}sin\varphi \right)}^{2}}} \end{array}$$

Expanding the first exponential term of $s\left(K_{r},K_{a}\right)$ into Taylor series up to the second order term with respect to ΔK=0, we have:

(9) $$\begin{array}{c} s\left(K_{r},K_{a}\right)\approx rect\left(\frac{K_{r}-K_{c}}{4\pi \alpha }\right)rect\left(\frac{K_{a}-K_{r}sin\varphi }{B_{a}}\right)exp\left(-j\phi_{0}-j\phi_{1}\Delta K-j\phi_{2}\Delta K^{2}\right)exp\left(-j\phi \left(X^{s}\right)\;\right) \end{array}$$

and:

(10) $$\phi_{0}=R_{0}cos\varphi \sqrt{{K_{rc}}^{2}-{\left(K_{a}-K_{rc}sin\varphi \right)}^{2}}-\left(K_{a}-K_{rc}sin\varphi \right)sin\varphi R_{0}-K_{rc}sin\varphi x_{n}$$

(11) $$\phi_{1}=R_{0}cos\varphi \frac{K_{rc}+\left(K_{a}-K_{rc}sin\varphi \right)sin\varphi }{\sqrt{{K_{rc}}^{2}-{\left(K_{a}-K_{rc}sin\varphi \right)}^{2}}}+R_{0}sin\varphi^{2}-x_{n}sin\varphi -\left(K_{a}-K_{rc}sin\varphi \right)\frac{v_{x}c}{4\pi \alpha \beta }$$

(12) $$\begin{array}{c} \phi_{2}\approx -R_{0}cos\varphi \frac{{K_{a}}^{2}}{2{\left({K_{rc}}^{2}-{\left(K_{a}-K_{rc}sin\varphi \right)}^{2}\right)}^{\frac{3}{2}}}+\frac{v_{x}csin\varphi }{4\pi \alpha } \end{array}$$

$\phi_{0}$ is the phase modulation term, $\phi_{1}$ is the RCM term in which the last term is introduced by intra-pulse motion, and $\phi_{2}$ is the SRC term accounting for additional chirp modulation with the last term which is introduced by intra-pulse motion after squint minimization. From the above formulas, we can see that RCM and range chirp modulation are largely reduced; however, both phase modulation term and RCM term are related to x-coordinate of the scatterers. So, handling them with the usual RCM correction (RCMC) and azimuth compression terms will only fully focus the scatterer at scene center, and result in residual RCM and phase error for scatterers away from scene center.

Therefore, in this paper, we first correct RCM with respect to scene center, then divide the coarsely RCM-corrected and focused data into azimuth sub-images and correct the residual RCMs with respect to their own scene centers with

(13) $$\begin{array}{cc} \phi_{1\_n}= & -\delta R_{n}cos\varphi \frac{K_{rc}-\left(K_{a}-K_{rc}sin\varphi \right)sin\varphi }{\sqrt{{K_{rc}}^{2}-{\left(K_{a}-K_{rc}sin\varphi \right)}^{2}}}-\delta R_{n}sin^{2}\varphi \end{array}$$

where $\delta R_{n}=-x_{n}sin\varphi$, which can be determined from zeroth order Taylor expansion of $\phi_{1}$. Block length should be properly selected, so that residual RCM of scatterers within one sub-image can be ignored. The advantages of this scheme are, firstly, maximum residual RCM is the same for all the sub-images no matter how far they are from the scene center; and secondly, no approximation is assumed to model the azimuth dependency of the Doppler parameters, so that even if there is residual RCM, it can be exactly known and easily controlled for the entire scene.

Residual phase differences can be handled with ANCS to avoid image discontinuities, and equalizes Doppler parameters of scatterers in the same range gate with a perturbation function in the azimuthal space domain.

## 4. Motion Compensation

Small-sized UAV platforms can easily deviate from its planned trajectory due to wind turbulences and/or unbalanced motor power delivery, which usually leads to large variance of the Doppler centroid:

(14) $$f_{dc}=\frac{-2\nabla \left(\frac{\left(P_{0}-A\right)}{∥P_{0}-A∥}\cdot \left(d+v_{\Delta }t_{m}+\frac{1}{2}a_{\Delta }{t_{m}}^{2}\right)\right)}{\lambda }-\frac{2v_{x}sin\varphi }{\lambda }$$

where λ is the wavelength. This can be resolved with a Doppler tracker indicated by ① in the flowchart, which determines Doppler centroid by identifying peak locations of their frequency spectrum. While the performance of Doppler tracker is subject to many factors and thus unable to obtain highly accurate estimations, it is still accurate enough to reduce image defocus by a large extent. After which, a more sophisticated algorithm can be used to obtain more refined focusing.

### 4.1. Squinted Azimuth-Dependent PGA

After squint minimization and the above two-stage RCMC, $s\left(R,X\right)$ can be expressed as:

(15) $$\begin{array}{c} s\left(R,X,x_{i}\right)=sinc\left(R-R_{0}\right)rect\left(X\right)\\ exp\left(\begin{array}{c} -jK_{rc}\sqrt{\begin{array}{c} {\left(\left(R_{0}+\left(x_{n}+x_{i}\right)sin\varphi \right)cos\varphi \right)}^{2}+\\ {\left(X+\left(R_{0}+\left(x_{n}+x_{i}\right)sin\varphi \right)sin\varphi -x_{i}\right)}^{2} \end{array}}+jK_{rc}Xsin\varphi -j\phi \left({K_{a}}_{c}^{s}\right)\; \end{array}\right) \end{array}$$

for a general scatterer $\left(R_{0},x_{i}\right)$ in the *n*’th subaperture having residual NsRCM not exceeding one range cell, where ${K_{a}}_{c}^{s}=K_{rc}\sin\varphi -K_{rc}\frac{X-x_{n}+R_{0}\sin\varphi }{\sqrt{{\left(X-x_{n}+R_{0}\sin\varphi \right)}^{2}+{\left(R_{0}\cos\varphi \right)}^{2}}}$ is the point of stationary phase, and $x_{n}$ denote azimuthal coordinate of the subaperture center.

After deramping with

(16) $$s_{deramp}=exp\left(\begin{array}{c} -jK_{rc}\sqrt{\begin{array}{c} {\left(\left(R_{0}+x_{n}sin\varphi \right)cos\varphi \right)}^{2}+\\ {\left(X+\left(R_{0}+x_{n}sin\varphi \right)sin\varphi \right)}^{2} \end{array}}+jK_{rc}Xsin\varphi \end{array}\right)$$

we have,

(17) $$\begin{array}{c} s\left(R,X,x_{i}\right)=sinc\left(R-R_{0}\right)rect\left(X\right)exp\left(-jA\left(x_{i}\right)-jB\left(x_{i}\right)X-jC\left(x_{i}\right)X^{2}-jO\left(X^{3}\right)\right) \end{array}$$

where

(18) $$A\left(x_{i}\right)=K_{rc}\sqrt{\begin{array}{c} {\left(\left(R_{0}+\left(x_{n}+x_{i}\right)sin\varphi \right)cos\varphi \right)}^{2}+\\ {\left(\left(R_{0}+\left(x_{n}+x_{i}\right)sin\varphi \right)sin\varphi -x_{i}\right)}^{2} \end{array}}-K_{rc}\left(R_{0}+x_{n}sin\varphi \right)$$

(19) $$B\left(x_{i}\right)=K_{rc}\frac{\left(R_{0}+\left(x_{n}+x_{i}\right)sin\varphi \right)sin\varphi -x_{i}}{\sqrt{\begin{array}{c} {\left(\left(R_{0}+\left(x_{n}+x_{i}\right)sin\varphi \right)cos\varphi \right)}^{2}+\\ {\left(\left(R_{0}+\left(x_{n}+x_{i}\right)sin\varphi \right)sin\varphi -x_{i}\right)}^{2} \end{array}}}-K_{rc}sin\varphi$$

(20) $$C\left(x_{i}\right)=K_{rc}\frac{{\left(\left(R_{0}+\left(x_{n}+x_{i}\right)sin\varphi \right)cos\varphi \right)}^{2}}{{\left(\begin{array}{c} {\left(\left(R_{0}+\left(x_{n}+x_{i}\right)sin\varphi \right)cos\varphi \right)}^{2}+\\ {\left(\left(R_{0}+\left(x_{n}+x_{i}\right)sin\varphi \right)sin\varphi -x_{i}\right)}^{2} \end{array}\right)}^{\frac{3}{2}}}-K_{rc}\frac{cos^{2}\varphi }{R_{0}+x_{n}sin\varphi }$$

From which we can see that, the second order term can hardly be canceled when squint angle is not zero. Exact deramping can only be achieved for the center scatterer, and azimuth dependent shift and defocusing still exist for scatterers other than the center one. This inconsistency can be attributed to, firstly, the aforementioned azimuth dependency of the Doppler parameters, and secondly, the nonlinearity of the difference systems formed by the phase of the deramping function, which is sderampX−sderampX−X0≠aX0X+b. The inexact deramping will affect motion error estimation, making it hard to combine motion error estimate from more than one scatterer. So that, in order for more accurate estimate, inexact deramping induced defocusing should be eliminated prior to motion error combination.

We thus resort to using the azimuthal locations in the pseudo-image after azimuth FFT. Suppose that the selected prominent scatterer is centering at the frequency bin $K_{ac}$, so that we have

(21) $$B\left(x_{i}\right)=K_{ac}$$

and

(22) $$\begin{array}{c} x_{i}=\frac{-2b+\sqrt{b^{2}-4ac}}{4a} \end{array}$$

where

(23) $$\left\{\begin{array}{c} a={\left(K_{ac}+sin\varphi \right)}^{2}\left(1+sin^{2}\varphi \right)-cos^{4}\varphi \\ b=2R_{0}sin\varphi cos^{2}\varphi \\ c=\left({\left(K_{ac}+sin\varphi \right)}^{2}-sin^{2}\varphi \right){R_{0}}^{2} \end{array}\right.$$

As soon as we have the azimuthal coordinate of the scatterer, exact deramping function can be constructed as

(24) $$\begin{array}{c} s\left(R,X,x_{i}\right)=exp\left(\begin{array}{c} -jK_{rc}\sqrt{\begin{array}{c} {\left(\left(R_{0}+\left(x_{n}+x_{i}\right)sin\varphi \right)cos\varphi \right)}^{2}+\\ {\left(X+\left(R_{0}+\left(x_{n}+x_{i}\right)sin\varphi \right)sin\varphi -x_{i}\right)}^{2} \end{array}}+jK_{rc}Xsin\varphi \end{array}\right) \end{array}$$

and then defocusing can be eliminated. This procedure can be iterated to improve the estimation accuracy of the azimuthal coordinate of the scatterers.

In the above Squinted Azimuth-dependent PGA (SAPGA) based MoCo scheme, SAPGA is applied to sub-apertures to obtain phase error estimation, refined by MEA, and then filtered and concatenated to full-aperture length. The whole procedure is indicated by ② in the flowchart. The reasons to refine SAPGA estimate with MEA is that, SAPGA still relies on the scene content for prominent scatterers to focus on, while MEA performs well even in natural scenes. Furthermore, the latter converges quickly when fed with the relatively accurate initial value provided by SAPGA. Once phase error is available, first order MoCo can be conducted by delay adjustment and phase correction, exploiting the analytical relationship between phase error and NsRCM. Furthermore, as imaging swath is relatively small in low attitude UAV SAR, the number of prominent scatterers in each sub-aperture is not large enough, so that accurate range dependent estimation can be hardly realized. Therefore, we divide the sub-apertures into slightly overlapping range blocks, and apply the aforementioned MoCo method to each range block to account for range dependencies, which is indicated by ③ in the flowchart.

### 4.2. NsRCM

The analytical relationship between phase error and residual NsRCM can be deduced by inspecting Equations (6) and (7), where $\phi \left(X^{s}\right)$ comprises phase error term and residual RCM term, which can be obtained through first order Taylor expansion with respect to ΔK=0 and expressed as (see Appendix A):

(25) $$\begin{array}{cc} \phi \left(X^{s}\right) & =K_{r}\Delta R\left(X^{s}\right)\\ & \begin{array}{c} =K_{rc}\Delta R\left(X_{\Delta K_{r}=0}^{s}\right)+\Delta K_{r}\cdot \left(\Delta R\left(X_{\Delta K_{r}=0}^{s}\right)+K_{rc}{\left(\frac{\partial \Delta R\left(X^{s}\right)}{\partial X^{s}}\frac{\partial X^{s}}{\partial \Delta K_{r}}\right)}_{\Delta K_{r}=0}\right) \end{array}\\ & \begin{array}{c} \approx K_{rc}\Delta R\left(X_{0}^{s}\right)+\Delta K_{r}\cdot \left(\Delta R\left(X_{0}^{s}\right)+\left(R_{0}\sin\varphi -\frac{R_{0}\sin\varphi }{\cos\varphi^{2}}\right)\frac{\partial \Delta R\left(X_{0}^{s}\right)}{\partial X_{0}^{s}}\right) \end{array} \end{array}$$

where $X_{0}^{s}=X_{\Delta K_{r}=0}^{s}$. The above approximation holds when azimuth bandwidth is relatively small and squint angle is not too large, in agreement with our sub-aperture-based SAPGA motion estimation scheme. As ${K_{a}}_{c}^{s}$ is a one to one mapping to *X* with no ΔK term involved, this relationship is maintained even after transforming into azimuth spatial domain. It is obvious that, residual RCM is slightly magnified following the processing procedure. However, the amount of magnification is far smaller than that of direct squinted wave number domain processing [23]. Furthermore, the inconsistency between phase error and NsRCM can be circumvented if range compression is done before transforming to the azimuthal space spectrum domain; however, a more time consuming interpolating scheme is needed to handle the SRC. For MoCo, this inconsistency should be taken into account when delay adjustment is done after range compression.

**Figure 2:**
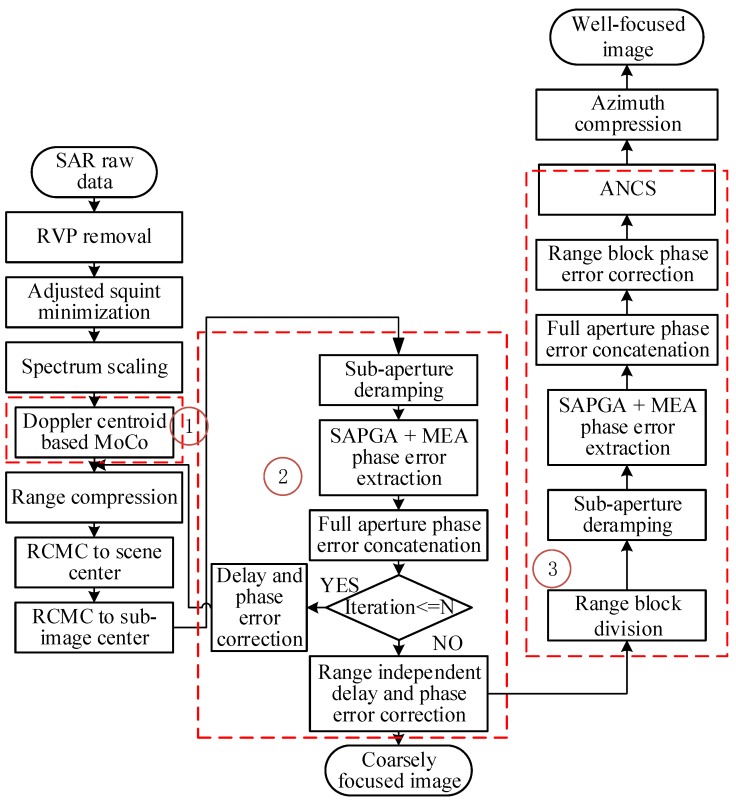
Flowchart of the proposed MoCo scheme.

The flowchart of the overall MoCo scheme is shown below in Figure 2, where MoCo steps are in the red dotted boxes and *N* is the maximum number of iteration. The majority of the imaging and motion compensation steps are quite fast. The only potential bottleneck is the MEA step whose computational complexity analysis can be found in [24]. In the MEA step, most of the time is spent in gradient vector and Hessian matrix computation as well as Fast Fourier Fransform (FFT) operations transforming the data between image and phase history domains. As the size of gradient vector, Hessian matrix, and FFT operation is directly proportional to the length of the processing aperture, the choice of the small-aperture-based autofocus strategy will also help to improve the computational efficiency. Moreover, the maximum number of iteration is set to 2, which is observed to be sufficient for obtaining satisfactory focusing results while saving computational time.

## 5. Simulations and Real Data Experiments

Both simulations and real data experiments are provided in this section to validate the proposed imaging and MoCo methods. Parameters of simulation and real data experiments are given in Table 1.

### 5.1. Simulations

**Figure 3:**
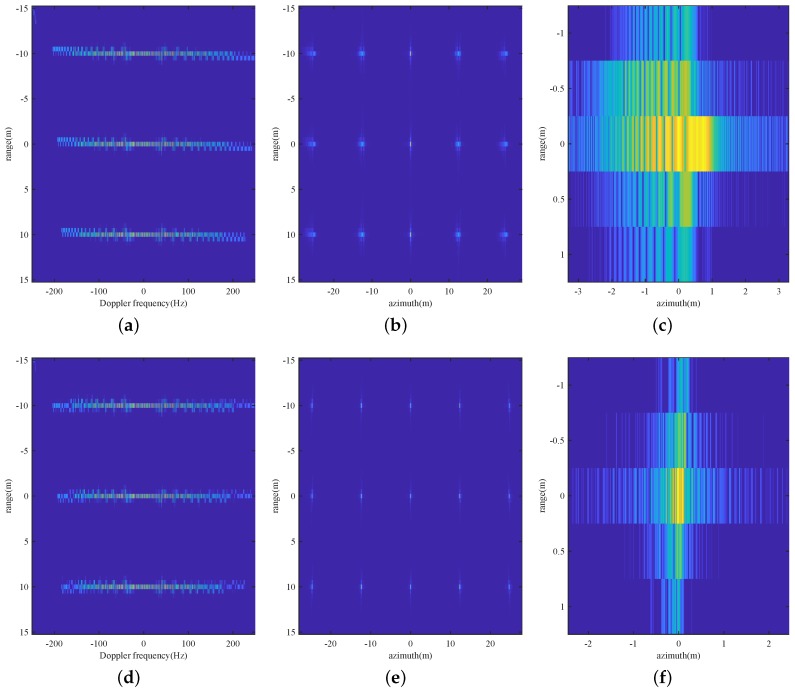
Point target verification of the imaging algorithm: (**a**) range profile without two-stage RCMC, (**b**) focused image without two-stage RCMC and ANCS, (**c**) intensity in dB of the scatterer in the left bottom corner without two-stage RCMC and ANCS, (**d**) range profile after two-stage RCMC, (**e**) focused image with two-stage RCMC and ANCS, (**f**) intensity in dB of the scatterer in the left bottom corner after two-stage RCMC and ANCS.

Firstly, 15 point scatterers separated by 12.5 m in azimuth and 10 m in range are simulated to verify the effectiveness of the imaging algorithm, whose results are given in Figure 3.

As demonstrated in Figure 3a–c, residual RCM still exceeds one range cell after RCMC with respect to the azimuth center, causing the image to be defocused, the further the scatterers are from the azimuth center, the more defocused they are. However, after the second stage RCMC, residual RCM are fully corrected, and the azimuth dependent Doppler parameters are successfully equalized, resulting in a focused image for scatterers all over the scene (Figure 3d–f).

### 5.2. Real Data Experiment

**Figure 4:**
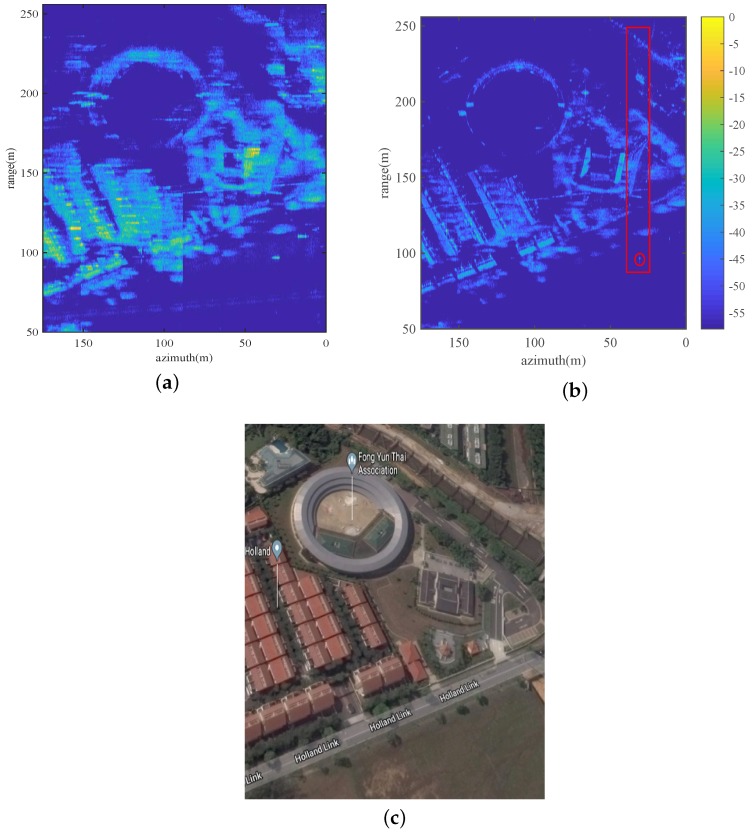
Real data experiments: (**a**) focusing result without MoCo, (**b**) fully focused SAR image, (**c**) optical image of trial scene from Google Map.

The flight trial was carried out at an urban area in Holland Link, Singapore. Optical image of the trial scene from Google Map is shown in Figure 4c as ground truth for comparison. The SAR data were collected by a radar mounted on a octal-rotor UAV flying at an attitude around 68 m during an overall collecting time of 60 s. Trajectory data provided by INS was of low accuracy and low sampling frequency according to our observation and thus left unused. The proposed imaging and MoCo algorithms were then directly applied.

**Figure 5:**
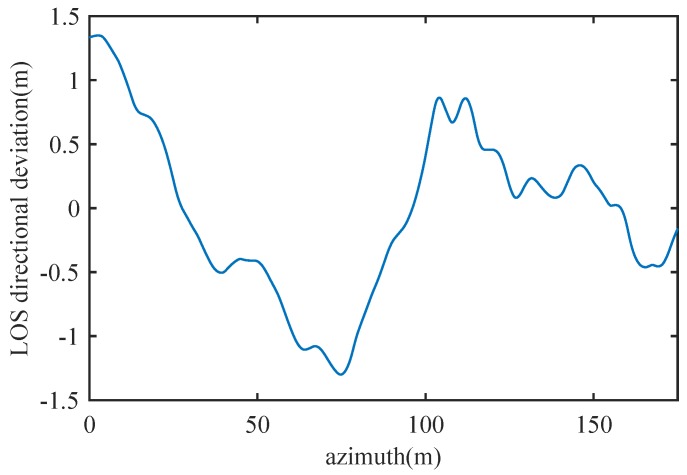
Trajectory deviation estimated with Doppler tracker.

**Figure 6:**
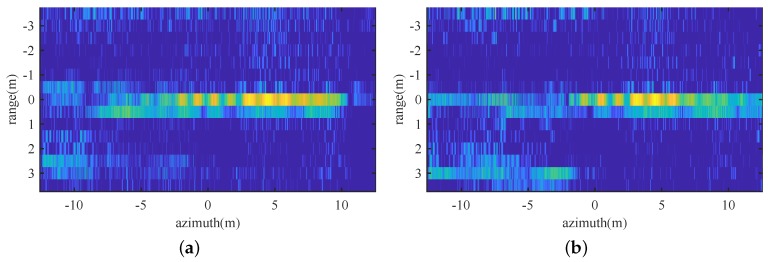
Range profiles: (**a**) before MoCo, (**b**) after MoCo.

Firstly, Doppler centroid estimation was carried out for platform stablization. The estimated trajectory errors were range-compensated. Ligh-of-sight (LOS) directional deviations estimated with Doppler Tracker is shown in Figure 5, from which we can see that deviations of up to 1.5 m were experienced during the collection, which is obviously large enough to destroy a Ka band SAR image. Range profiles before and after MoCo are shown in Figure 6, it is clear that both azimuth dependent RCM and residual erroneous RCM are fully corrected.

SAPGA and MEA-based MoCo approach described above was then applied. Focusing results before and after MoCo are shown for comparisons in Figure 4a,b. As the trajectory deviation was rather significant, large amount of blurring and distortions are present in Figure 4a, making the uncompensated image unusable. On the other hand, the imaging quality has been greatly improved after SAPGA and MEA-based MoCo. The hall in the center of the image appears more rounded, skeletons of the blocks are clearer, as well as the trees and bushes around buildings. It is worth mentioning that, roofs illuminated by the radar is squeezed and those not illuminated are stretched due to their relative slop angle interacting with the radar beam, resulting in large area of shadows between blocks.

**Figure 7:**
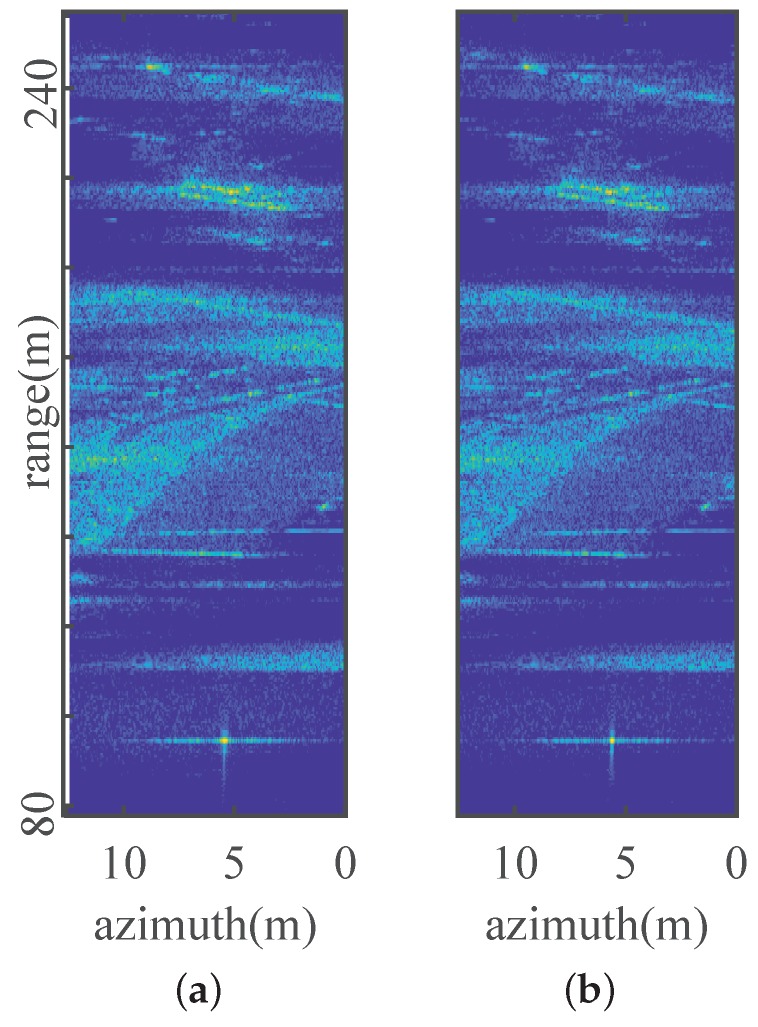
Local area autofocused using: (**a**) PGA, (**b**) SAPGA.

**Figure 8:**
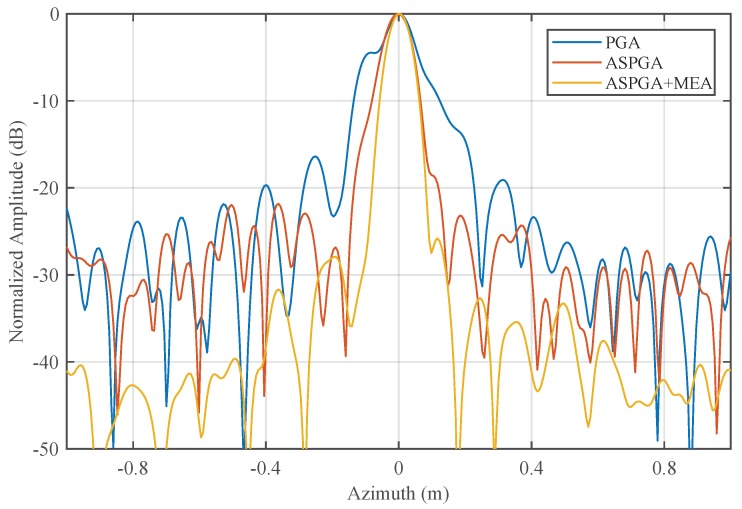
Azimuth responses of strong scatterer at the right bottom corner.

Weighted non-iterative PGA [13,14] is used for comparison to verify the effectiveness of SAPGA. Local areas indicated in the red box in Figure 4b is autofocused with PGA and SAPGA whose results are given in Figure 7. As we can see, edges of the roofs are clearer, and contrast is also enhanced after exact deramping with SAPGA in Figure 7b. For a more quantitative comparison, the azimuth response of a corner reflector located in the red circle are given in Figure 8. Focusing of the corner reflector has improved a lot in terms of both main-lobe width and side-lobes level with the proposed algorithm. Entropies of the focused images using PGA and SAPGA are 10.7946 and 10.7488 respectively, showing a small improvement. On the other hand, the main-lobe widths is reduced from 0.38 to 0.25 m and further reduced to 0.2 m after refinement using MEA. All the results indicate superior performance of SAPGA over PGA after squint minimization, and effectiveness of our proposed imaging and MoCo algorithms.

## 6. Conclusions

To process small-squinted UAV-borne SAR data, we have proposed a signal processing approach which effectively focuses the data and achieved high resolution SAR imaging. The proposed signal processing approach accomplishes squint mode imaging and motion compensation for FMCW SAR by decreasing its impact on the processing procedure, which largely simplifies the focusing workflow and opens the potential for larger squinted imaging. A PGA variation called SAPGA is also proposed, which successfully handles azimuth dependent Doppler parameter and inexact deramping problems and realizes accurate data-driven motion compensation. The proposed imaging and motion compensation algorithm eases mission design considerations for small UAV-bore SAR platforms and saves cost on highly accurate INS instruments and high-end hardware, making UAV-bore SAR good choices as test-bed for many modern SAR applications.

As imaging is achievable in monostatic UAV-bore SAR, our future work will be towards repeated path interferometric SAR to obtain altitude information about the imaging scene, thus realize 3D imaging on small platforms. Furthermore, once stationary clutters are eliminated, moving target indication (MTI) can be performed, which is also important for modern UAV-bore SAR applications. The availability of altitude information, on the other hand, will improve the accuracy of MTI, thus towards a highly accurate and cost efficient 3D imaging and MTI system. 

## Figures and Tables

**Table 1:** UAVSAR Parameters.

Carrier frequency	35.075 GHz	Bandwidth	300 MHz
velocity	around 3 m/s	Attitude	around 68 m
PRF	500 Hz	Squint angle	10 degrees
Reference range	158 m	Elevation angle	64 degrees

## Nomenclature

*H*	platform attitude	$t_{d}$	delay of the transmitted wave
$R_{0}$	slant range distance at synthetic aperture center	$f_{c}$	carrier frequency
φ	squint angle	α	chirp rate
$v_{x}$	platform velocity	$K_{r}$	slant range directional wavenumber
$K_{a}$	azimuthal wavenumber	*c*	speed of light
*t*	fast time	$t_{m}$	slow time

## Appendix A. NsRCM

According to Equation (23)

(A1) $$\begin{array}{c} \phi \left(X^{s}\right)=K_{r}\Delta R\left(X^{s}\right)\\ \begin{array}{c} =K_{rc}\Delta R\left(X_{\Delta K_{r}=0}^{s}\right)+\Delta K_{r}\cdot \\ \;\left(\Delta R\left(X_{\Delta K_{r}=0}^{s}\right)+K_{rc}{\left(\frac{\partial \Delta R\left(X^{s}\right)}{\partial X^{s}}\frac{\partial X^{s}}{\partial \Delta K_{r}}\right)}_{\Delta K_{r}=0}\right) \end{array} \end{array}$$

where

(A2) $$\begin{array}{cc} \frac{\partial X^{s}}{\partial \Delta K_{r}} & =\begin{array}{c} -\frac{v_{x}\cdot c}{4\pi \alpha \beta }\\ +\frac{R_{0}cos\varphi sin\varphi }{{\left(K_{r}^{2}-{\left(K_{r}sin\varphi -K_{a}\right)}^{2}\right)}^{\frac{1}{2}}}\\ \begin{array}{c} +R_{0}cos\varphi \left(K_{r}sin\varphi -K_{a}\right)\cdot \left(-\frac{1}{2}\right)\cdot \\ \left[\frac{2K_{r}-2\left(K_{r}sin\varphi -K_{a}\right)sin\varphi }{{\left(K_{r}^{2}-{\left(K_{r}sin\varphi -K_{a}\right)}^{2}\right)}^{\frac{3}{2}}}\right] \end{array} \end{array}\\ & \approx \begin{array}{c} -\frac{v_{x}\cdot c}{4\pi \alpha \beta }+\frac{R_{0}cos\varphi sin\varphi }{K_{r}cos\varphi }\\ +\frac{R_{0}cos\varphi {K_{r}}^{2}{sin\varphi }^{3}-{K_{r}}^{2}sin\varphi }{{K_{r}}^{3}{cos\varphi }^{3}} \end{array} \end{array}$$

by neglecting influences of $K_{a}$ and ignoring high order terms of ${sin\varphi }^{3}$, then we arrive at

(A3) $$\begin{array}{c} \phi \left(X^{s}\right)=K_{r}\Delta R\left(X^{s}\right)\\ \begin{array}{c} \approx K_{rc}\Delta R\left(X_{0}^{s}\right)+\Delta K_{r}\cdot \\ \left(\;\Delta R\left(X_{0}^{s}\right)+\left(R_{0}\sin\varphi -\frac{R_{0}\sin\varphi }{\cos\varphi^{2}}\right)\frac{\partial \Delta R\left(X_{0}^{s}\right)}{\partial X_{0}^{s}}\right) \end{array} \end{array}$$

thus Equation (23). So that should the above analysis be held true, processed azimuth bandwidth must be kept small and squint angle not too large, making it suitable for sub-aperture-based processing.
